# Epidemiology, radiology, and genetics of nicotine dependence in COPD

**DOI:** 10.1186/1465-9921-12-9

**Published:** 2011-01-13

**Authors:** Deog Kyeom Kim, Craig P Hersh, George R Washko, John E Hokanson, David A Lynch, John D Newell, James R Murphy, James D Crapo, Edwin K Silverman

**Affiliations:** 1 Channing Laboratory, Brigham and Women's Hospital, Boston, MA, USA; 2 Division of Pulmonary and Critical Care Medicine, Seoul National University Boramae Medical Center, Seoul, South Korea; 3 Division of Pulmonary and Critical Care Medicine, Brigham and Women's Hospital, Boston, MA, USA; 4 Colorado School of Public Health, University of Colorado Denver, Aurora, USA; 5 Department of Radiology, National Jewish Health, Denver, CO, USA; 6 Department of Biostatistics, National Jewish Health, Denver, CO, USA; 7 Department of Medicine, National Jewish Health, Denver, CO, USA

## Abstract

**Background:**

Cigarette smoking is the principal environmental risk factor for developing COPD, and nicotine dependence strongly influences smoking behavior. This study was performed to elucidate the relationship between nicotine dependence, genetic susceptibility to nicotine dependence, and volumetric CT findings in smokers.

**Methods:**

Current smokers with COPD (GOLD stage ≥ 2) or normal spirometry were analyzed from the COPDGene Study, a prospective observational study. Nicotine dependence was determined by the Fagerstrom test for nicotine dependence (FTND). Volumetric CT acquisitions measuring the percent of emphysema on inspiratory CT (% of lung <-950 HU) and gas trapping on expiratory CT (% of lung <-856 HU) were obtained. Genotypes for two SNPs in the CHRNA3/5 region (rs8034191, rs1051730) previously associated with nicotine dependence and COPD were analyzed for association to COPD and nicotine dependence phenotypes.

**Results:**

Among 842 currently smoking subjects (335 COPD cases and 507 controls), 329 subjects (39.1%) showed high nicotine dependence. Subjects with high nicotine dependence had greater cumulative and current amounts of smoking. However, emphysema severity was negatively correlated with the FTND score in controls (ρ = -0.19, p < .0001) as well as in COPD cases (ρ = -0.18, p = 0.0008). Lower FTND score, male gender, lower body mass index, and lower FEV1 were independent risk factors for emphysema severity in COPD cases. Both CHRNA3/5 SNPs were associated with FTND in current smokers. An association of genetic variants in CHRNA3/5 with severity of emphysema was only found in former smokers, but not in current smokers.

**Conclusions:**

Nicotine dependence was a negative predictor for emphysema on CT in COPD and control smokers. Increased inflammation in more highly addicted current smokers could influence the CT lung density distribution, which may influence genetic association studies of emphysema phenotypes.

**Trial registration:**

ClinicalTrials (NCT): NCT00608764

## Introduction

Cigarette smoking is the most important environmental risk factor for the development of COPD [1-3]. Cigarette smoking intensity is known to be associated with clinical features of COPD such as the rate of lung function decline [2,4] and COPD exacerbation frequency [5,6]. In addition, it is correlated with symptoms of chronic bronchitis even in healthy smokers [7]. However, the correlation between the amount of lifetime smoking measured as pack-years and the severity of emphysema on chest CT scans is weak [8,9]. Although the extent of exposure to cigarette smoke is usually measured in pack-years, this metric does not reflect other aspects of smoking behaviors such as depth of inhalation, number of puffs per cigarette, and age of onset of smoking [10]. Nicotine dependence develops in many smokers, and smokers with dependency to nicotine tend to have increased smoking intensity [11]. Thus, nicotine dependence may increase the impact of smoking exposure due to altering the frequency or depth of smoke inhalation, even in COPD patients with the same pack-year history. As a result, it is reasonable to hypothesize that increased dependence to nicotine would facilitate the development and progression of COPD. Detailed phenotyping of COPD includes measures of emphysema severity and air trapping determined by quantitative analysis of chest CT scans. These radiographic measurements have been correlated with respiratory symptoms and physiologic parameters [12,13]. Despite the highly plausible connection, there have not been reports documenting an association between nicotine dependence and the radiographic features of COPD.

It has been reported that nicotine addiction is influenced by alpha-nicotinic acetylcholine receptor (nAChR) variants on chromosome 15 [10,11,14,15]. This genetic locus is also associated with lung cancer [16,17] and has been reported as one of the major susceptibility loci identified by a genome-wide association study (GWAS) in COPD [14]. To understand the relationship of this genetic locus to COPD susceptibility, it is important to understand its impact on nicotine dependence and COPD-related phenotypes. Recently, it was reported that the 15q25 locus of the nAChR (CHRNA3/5) was associated with presence and severity of emphysema, but an association of this genetic locus with nicotine addiction measured by the number of pack-years was not found, and more than half of the subjects were ex-smokers [18]. Therefore, we assessed the association of nicotine dependence measured by the Fagerstrom test for nicotine dependence (FTND), SNPs previously related to nicotine dependence on chromosome 15, and the severity of radiographic features of COPD including emphysema and gas-trapping. We hypothesized that increased nicotine dependence would be correlated with increased radiographic severity of COPD independent of pack-years of smoking and that the genetic susceptibility locus on chromosome 15 would be associated with nicotine dependence and the radiographic features of COPD.

## Methods

### Subjects

The Genetic Epidemiology of COPD (COPDGene) Study (http://www.copdgene.org/) is a multicenter prospective observational study designed to identify genetic factors associated with COPD and to characterize COPD-related phenotypes [19]. This study recruited COPD cases and smoking controls who were non-Hispanic whites and African Americans ages 45 to 80 with at least 10 pack-years of smoking history. This analysis is based on the first 2500 eligible subjects enrolled into COPDGene at 21 clinical centers across the United States (April 2010 COPDGene data set). In this analysis, COPD was defined by GOLD stage ≥ II criteria of post-bronchodilator FEV1 <80% predicted and FEV1/FVC ratio <0.7[3]. Subjects with GOLD stage 1 or GOLD-unclassified (FEV1 <80% predicted and FEV1/FVC >0.7) were excluded to focus our investigation on a comparison of COPD subjects with definite reductions in FEV1 and smoking controls.

The Fagerstrom Test for Nicotine Dependence (FTND) [20,21] was included in the COPDGene study questionnaires to assess nicotine dependence in current smokers only. The detailed enrollment criteria and phenotyping methods in the COPDGene study have been described elsewhere [19]. This analysis included only the COPD cases and controls who were current smokers at study enrollment with available FTND scores. In the analysis of genetic association with emphysema, ex-smokers were also tested to assess the differences of the association evidence according to current smoking status.

### CT imaging

Using multi-detector CT scanners with at least 16 detector channels, an inspiratory chest CT at 200 mAs was performed to assess for percent of emphysema (% of lung <-950 HU) and an expiratory chest CT at 50mAs was performed to measure air trapping (% of lung <-856 HU). Lung volumes including total lung capacity (TLC) and functional residual capacity (FRC) were also determined from the chest CT scans using 3D-SLICER software (http://www.slicer.org). Since Siemens Sensation 64 CT scanners with the B31f reconstruction kernel had an outlier pattern of lung density distribution, we included a covariate for CT scans performed on that platform. The extent of emphysema was classified in four groups according to the percent of emphysema: none or trivial (<5% of lung involved), mild (5-25%), moderate (25-50%), or severe (>50%) [22]. Airway wall area at a lumen perimeter of 10 mm and quantitative emphysema measurement have been reported to contribute independently to airflow obstruction in COPD [22]. Airway wall area was estimated from the relationship between square root of airway wall area and airway luminal perimeter for a hypothetical 10 mm luminal perimeter (Pi_10_) airway using VIDA [23,24]. In this analysis, Pi_10 _data were available in 359 control subjects (70.8%) and 261 COPD cases (77.9%).

### SNP genotyping

The A-allele for rs1051730 in the CHRNA3/5 locus has been associated with reduced FEV1, reduced diffusing capacity, and increased risk of emphysema on CT [18], and the C-allele for rs8034191 has been reported to increase the susceptibility to COPD and lung cancer [14,25]. We tested the genotypes of these two SNPs in the CHRNA3/5 locus (rs8034191, rs1051730) on chromosome 15 using TaqMan genotyping in a subset of COPDGene subjects.

Detailed genotyping and quality control methods as well as the associations between these SNPs and COPD have been previously reported by our group [26]. Association analysis of these SNPs was limited to non-Hispanic White COPD cases and control subjects.

### Statistical analysis

The total score of FTND was used as a quantitative phenotype. For the qualitative analysis of severity of nicotine dependence, subjects were dichotomized into a group with nicotine dependence (high dependence, FTND ≥ 6) or not (no/low dependence, FTND ≤ 5). As percent of emphysema on chest CT was non-normally distributed, natural log-transformed values were calculated to improve the normality. As the number of subjects with available Pi_10 _values was limited, Pi_10 _was not included in multivariate analysis. Univariate analyses were done using the chi-square test for categorical variables and the student t-test or ANOVA for continuous variables. Cochran-Armitage trend test was also used for categorical variables. Significance of correlation was determined by Pearson coefficient and its p-value. For assessing the determinants of the severity of emphysema and gas trapping on chest CT, linear regression analysis was performed. In multivariate analyses, statistically significant variables from the univariate analyses and additional clinically relevant variables were inserted in the models as covariates. To test the association with SNPs and key phenotypes, an additive model with linear regression analysis for continuous variables was applied. Statistical analysis was done using SAS (version 9.1) and statistical significance was determined by a p-value < 0.05.

## Results

### Characteristics of study subjects

Eight hundred forty-two subjects out of the first 2500 COPDGene subjects were currently smoking COPD or control subjects with a complete FTND score. The baseline characteristics of 507 current smokers without airflow obstruction and 335 COPD subjects with GOLD stage ≥ II are listed in Table 1. COPD subjects were older in age, and white race was more common among COPD cases. As expected, COPD subjects showed more severe airflow obstruction than smoking controls with compatible radiographic findings. Emphysema severity and air-trapping were more severe, and airway wall area was greater in COPD subjects. Mean FTND score, percentage of cases with high nicotine dependence (FTND score ≥6), the age started smoking, and the current number of cigarettes smoked daily were not different between COPD cases and control subjects, although the total amount of smoking in pack-years and average number of cigarettes smoked per day were higher in COPD subjects. There was no difference in the number of cases tested with a Siemens Sensation-64 CT scanner (17.6% for controls vs. 18.5% for COPD, p = 0.72), which generally showed a higher score of emphysema severity.

**Table 1:** Baseline characteristics of currently smoking subjects*

Variables	Control (n = 507)	COPD (n = 335)	p
Age, years	53.8 ± 6.9	59.2 ± 7.8	<.0001
Male, n (%)	276(54.4)	185(55.2)	0.82
White, n (%)	245(48.3)	244(72.8)	<.0001
BMI, kg/m^2^	28.5 ± 5.9	27.1 ± 5.8	0.001
FTND score	4.6 ± 2.5	4.7 ± 2.4	0.49
Nicotine dependence (FTND ≥6), n (%)	196(38.7)	133(39.7)	0.76
Smoking amount, pack-years	37.2 ± 19.2	51.7 ± 28.8	<.0001
Average number of cigarette smoked per day	20.2 ± 8.9	23.8 ± 10.8	<.0001
Cigarettes/day, current	16.9 ± 9.8	17.3 ± 10.8	0.56
Smoking starting age, years	17.0 ± 5.3	16.4 ± 4.8	0.09
FEV1/FVC, ratio	0.79 ± 0.05	0.53 ± 0.12	<.0001
FEV1, % of predicted	97.6 ± 12.3	55.2 ± 16.4	<.0001
% Emphysema at -950 HU^‡^, %	1.84 ± 2.21	8.81 ± 9.75	<.0001
% Gas trapping, %	10.5 ± 9.9	32.9 ± 19.3	<.0001
Pi_10_	3.75 ± 0.12	3.81 ± 0.12	<.0001

*Data listed in number (%) for frequency or mean ± standard deviation for quantitative variables.

^† ^The number of cases evaluated with Siemens Sensation CT scanner with 64 channels.

^‡ ^For epidemiologic and genetic analysis, log transformed values were used.

### Clinical characteristics of study subjects by the severity of nicotine dependence

The clinical characteristics of subjects with low and high values for FTND are shown in Table 2. When the subjects were dichotomized according to the severity of nicotine dependence (No/low dependence vs. high dependence), there was no difference in the percentage of subjects with high nicotine dependence between control and COPD groups (Table 2). The percentages of subjects in six FTND severity classes were also not different between control and COPD subjects (Additional file 1: Table S1). However, a decreasing number of highly addicted COPD subjects was found with increasing GOLD stages (p = 0.004 for trend, Table 2 and Additional file 1: Figure S1). COPD subjects showed lower FEV1 (% predicted) and FEV1/FVC in the low nicotine dependence group. These findings might reflect efforts to reduce smoking intensity in more severely affected subjects. In controls, subjects with high nicotine dependence showed lower FEV1 (% predicted) than subjects with low nicotine dependence even though all values were within the normal range (p = 0.047). Subjects with low FTND scores were older than those with severe dependence. In an analysis of racial distributions, white race proportion was higher in the full set of subjects with high FTND than in subjects with low FTND (62.9% vs. 55.0%, p = 0.02). However, an elevated proportion of white subjects with high FTND was found only in the COPD group. In terms of smoking behaviors, the total amount of pack-years and the current number of cigarettes smoked daily were higher and smoking starting age was earlier in the group with severe dependence in both controls and COPD cases. Symptoms of chronic bronchitis were more prevalent in COPD subjects with high nicotine dependence, and a similar trend was found in controls (Table 2).

**Table 2:** Baseline characteristics classified by the presence of nicotine dependence

Variables		No/Low dependence (n = 513)	High dependence (n = 329)	p
Subjects	Control	311(61.3)	196 (38.7)	0.76
	COPD	202(60.3)	133(39.7)	

COPD stage	GOLD II	116(55.0)	95(45.0)	0.02
	GOLD III	66(66.7)	33(33.3)	
	GOLD IV	20(80.0)	5(20.0)	

*Spirometry*				
FEV1/FVC %	Control	0.79 ± 0.05	0.79 ± 0.05	0.25
	COPD	0.52 ± 0.13	0.54 ± 0.11	0.03
FEV1pred %	Control	98.5 ± 12.5	96.3 ± 11.9	0.047
	COPD	53.5 ± 17.2	57.8 ± 14.7	0.02

*Demographic*				
Age, year	Control	54.8 ± 7.4	52.3 ± 5.8	<.0001
	COPD	60.0 ± 8.0	57.9 ± 7.5	0.02
Male, n (%)	Control	158(50.8)	118(60.2)	0.04
	COPD	107(53.0)	78(58.7)	0.31
White, n (%)	Control	147(47.3)	98(50.0)	0.55
	COPD	135(66.8)	109(82.0)	0.002

*Smoking related*				
Pack-years of smoking	Control	32.2 ± 15.6	45.2 ± 21.5	<.0001
	COPD	45.8 ± 23.4	60.6 ± 33.7	<.0001
Average number of cigarettes smoked per day	Control	17.3 ± 7.4	24.8 ± 9.2	<.0001
	COPD	21.0 ± 8.7	28.1 ± 12.2	<.0001
Cigarettes/day, current	Control	12.7 ± 7.3	23.6 ± 9.7	<.0001
	COPD	12.4 ± 7.8	24.8 ± 10.5	<.0001
Smoking starting age, years	Control	17.5 ± 5.5	16.2 ± 4.9	0.01
	COPD	17.0 ± 4.9	15.5 ± 4.6	0.01

*Symptom*				
Chronic bronchitis symptoms*, n (%)	Control	49(15.8)	43(21.9)	0.08
	COPD	64 (31.7)	72(54.1)	<.0001

*Radiographic*				
% Emphysema at -950 HU, %	Control	2.1 ± 2.4	1.5 ± 1.9	0.01
	COPD	10.5 ± 10.8	6.2 ± 7.1	<.0001
% Gas trapping, %	Control	10.7 ± 9.9	10.1 ± 9.9	0.52
	COPD	36.0 ± 20.6	28.3 ± 16.3	0.001
Pi_10_	Control	3.75 ± 0.13	3.75 ± 0.11	0.72
	COPD	3.80 ± 0.12	3.83 ± 0.12	0.11

* Chronic bronchitis is defined as cough for 3 mos/yr for at least 2 years along with phlegm for 3 mos/yr for at least 2 years.

### The correlation between radiographic parameters and the severity of nicotine dependence

**Figure 1:**
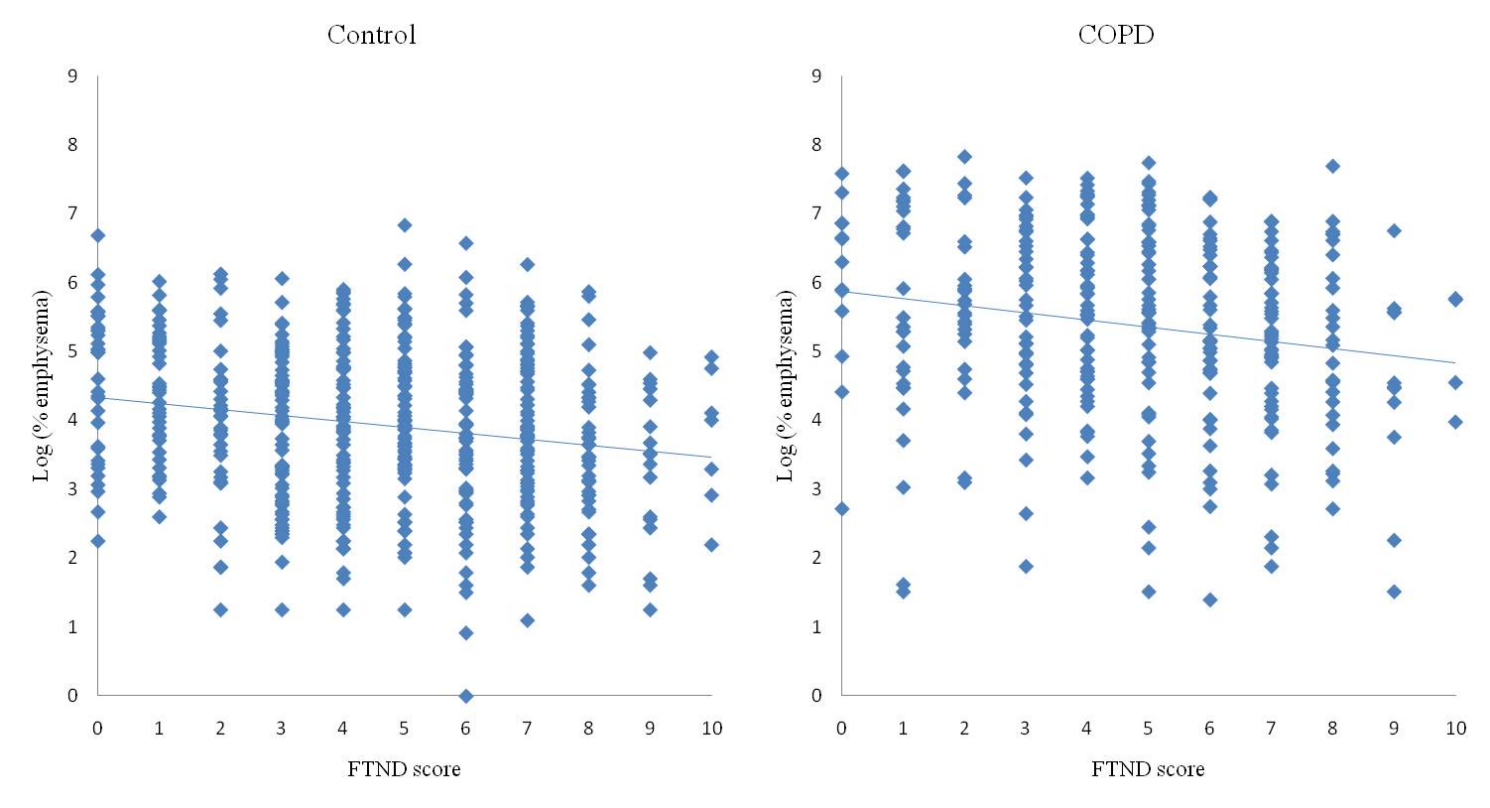
**The correlation of FTND score with emphysema severity on chest CT in controls (left; log (%emphysema) = 4.33-(0.09 × FTND score), p < .0001) and COPD subjects (right; log (%emphysema) = 5.87-(0.10 × FTND score), p = 0.0008)**.

In COPD subjects, the percent of emphysema and gas-trapping were greater in subjects with low FTND scores (Table 2). When COPD subjects were classified by the extent of emphysema, cases with more severe emphysema showed lower FTND scores (p = 0.001, Table 3). This relationship was also consistent when the FTND score was tested as a quantitative variable and the same pattern was also observed in controls (Figure 1). However, when comparisons were limited to subjects within a particular GOLD class, the statistical significance of negative correlations between FTND and emphysema was marginal (ρ = -0.13, p = 0.07 for subjects with GOLD II, ρ = -0.17, p = 0.06 for GOLD III-IV). In correlation analysis of FTND score with other radiographic variables including percent of gas-trapping and airway wall thickness measured on chest CT, percent of gas-trapping was significantly negatively correlated with FTND only in COPD cases (ρ = -0.19, p = 0.001). Airway wall thickness did not show any significant correlation with FTND score in COPD cases or control subjects. In terms of radiographic parameters, current smokers showed less extensive emphysema and gas trapping than ex-smokers in controls and COPD subjects although currently smoking COPD cases had less severe airflow obstruction than ex-smoking COPD cases (Additional file 1: Table S2). This finding was consistent for emphysema and gas trapping when COPD subjects were stratified within GOLD stages (GOLD II vs. GOLD III-IV).

**Table 3:** Differences in smoking variables by the extent of emphysema on chest CT in COPD subjects

	Extent of emphysema (%)*			
		
Variables	None/Trivial (<5%) (n = 167)	Mild (5-25%) (n = 134)	Moderate (25-50%) (n = 31)	P
FTND severity				0.0008^†^
No/low dependence	89(44.3)	85(42.3)	27(13.4)	
High dependence	78(59.5)	49(37.4)	4(3.0)	
FTND score	5.0 ± 2.4	4.7 ± 2.4	3.4 ± 2.0	0.001
Smoking starting age, years	16.6 ± 5.1	16.3 ± 4.6	15.6 ± 4.4	0.53
Pack-years of smoking	47.5 ± 22.2	57.6 ± 35.1	49.7 ± 27.7	0.01
Average number of cigarettes smoked per day	23.3 ± 9.2	25.0 ± 12.8	22.0 ± 9.4	0.24
Cigarettes/day, current	18.8 ± 10.2	16.9 ± 11.8	11.1 ± 7.3	0.001

*data of extent of % emphysema were missing in 3 subjects of COPD subjects.

^† ^p value for Fisher's Exact Test

### The determinants of the severity of emphysema and gas trapping

To determine the effect of FTND score on the severity of emphysema and gas trapping, multivariate analysis was done. In addition to FTND score, other significant predictors of emphysema and gas trapping from univariate analysis were included: age, gender, race, BMI, FEV1 (% predicted), type of CT scanner, and smoking intensity in pack-years (Table 4).

**Table 4:** The results of multivariate regression analysis for the severity of emphysema and gas-trapping*

	Log (% emphysema)				% gas-trapping			
Group	**COPD**		**Control**		**COPD**		**Control**	

variables	β	p	β	p	β	p	β	p

Age	0.01	0.17	0.01	0.06	0.23	0.046	0.32	<.0001
Female	-0.69	<.0001	-0.60	<.0001	-5.72	0.0003	-2.14	0.02
African-American	-0.10	0.51	-0.26	0.01	1.01	0.59	0.24	0.80
BMI, kg/m^2^	-0.06	<.0001	-0.01	0.12	-1.04	<.0001	-0.28	0.0003
FEV1pred%	-0.03	<.0001	0.01	0.002	-0.69	<.0001	-0.003	0.94
FTND score	-0.07	0.01	-0.07	0.0003	-0.62	0.09	-0.04	0.92
Pack-years of smoking	0.003	0.25	0.002	0.50	0.03	0.36	-0.02	0.49

*Additionally adjusted for CT scanner model.

In terms of the severity of emphysema, FTND score was as an independent predictor of emphysema despite adjusting for the total amount of smoking in pack-years but, as in the univariate analysis, the direction was negative rather than positive in controls as well as in COPD cases. Even when the severity of nicotine dependence was considered as a dichotomous variable instead of a quantitative FTND score, the statistical significance was consistent (β = -0.44, p = 0.002 for COPD and β = -0.27, p = 0.007 for control subjects).

When each multivariate model was adjusted for the number of daily cigarettes smoked currently instead of pack-years, the significance of FTND was persistent in only control subjects for severity of emphysema (β = -0.06, p = 0.008 for controls; β = -0.02, p = 0.57 for COPD) and the effect of FTND on gas trapping failed to reach statistical significance (β = 0.38, p = 0.12 for controls; β = -0.08, p = 0.86 for COPD). A significant interaction between FEV1 and FTND was not found.

### The association among candidate SNPs, nicotine dependence, and radiographic findings

Genotyping data for 312 currently smoking subjects with FTND scores (146 cases with COPD and 166 smoker controls) were available. In the full group of currently smoking subjects, the A-allele of rs1051730 was associated with increased FTND score, while neither SNP in the CHRNA3/5 region (rs8034191 and rs1051730) was associated with FEV1 (% predicted), affection status of COPD, nor percent of emphysema (Table 5). In stratified analysis, these SNPs were associated with increasing pack-years in currently smoking control subjects.

**Table 5:** The association with SNPs of CHRNA3/5 and nicotine dependence, lung function, and emphysema severity in current smokers

SNP (Chr. 15)		rs8034191				rs1051730			
Genotype (Number of subjects)		TT(126)	TC(145)	CC(38)	p	GG(129)	GA(137)	AA(36)	p

COPD affection		49(38.9)	78(53.8)	17(44.7)	0.13	50(38.8)	72(52.6)	16(44.4)	0.14

FTND score	Combined (312)	4.5 ± 2.4	5.0 ± 2.6	5.1 ± 2.6	0.12	4.5 ± 2.4	5.1 ± 2.7	5.3 ± 2.6	0.03
	COPD (146)	4.8 ± 2.2	5.1 ± 2.6	5.4 ± 2.2	0.35	4.7 ± 2.2	5.3 ± 2.6	5.1 ± 2.1	0.27
	Control (166)	4.3 ± 2.5	4.9 ± 2.7	4.8 ± 3.0	0.29	4.3 ± 2.5	4.8 ± 2.8	5.5 ± 2.9	0.08

Pack-years of smoking	Combined	46.2 ± 28.2	49.5 ± 24.2	47.1 ± 25.9	0.56	45.9 ± 27.8	49.7 ± 25.2	47.2 ± 24.6	0.46
	COPD	60.0 ± 36.5	55.8 ± 27.2	47.4 ± 26.3	0.16	59.3 ± 36.3	56.8 ± 28.7	45.4 ± 22.3	0.18
	Control	37.4 ± 16.1	42.1 ± 17.8	46.8 ± 26.2	0.02	37.4 ± 16.0	41.8 ± 17.8	48.7 ± 26.7	0.01

FEV1%pred	Combined	80.4 ± 25.0	72.1 ± 26.6	78.2 ± 24.5	0.14	80.2 ± 25.2	74.4 ± 25.5	77.6 ± 25.0	0.21
	COPD	54.1 ± 16.8	51.8 ± 18.4	56.4 ± 16.3	0.96	53.6 ± 16.9	54.6 ± 17.6	55.4 ± 17.7	0.69
	Control	97.1 ± 11.0	95.8 ± 9.9	95.9 ± 12.9	0.49	97.0 ± 10.9	96.4 ± 10.0	95.3 ± 12.7	0.50

Log(%emphysema)	Combined	4.7 ± 1.4	5.0 ± 1.3	4.6 ± 1.4	0.57	4.7 ± 1.4	4.9 ± 1.2	4.7 ± 1.5	0.60
	COPD	5.5 ± 1.2	5.6 ± 1.2	5.5 ± 1.4	0.87	5.6 ± 1.2	5.5 ± 1.2	5.8 ± 1.2	0.82
	Control	4.2 ± 1.2	4.3 ± 1.0	4.0 ± 1.0	0.78	4.2 ± 1.2	4.3 ± 1.0	3.9 ± 0.9	0.61

In multivariate models, addition of either the A-allele of rs1051730 or the C-allele of rs8034191 was significantly associated with increased FTND score independent of age and gender in the full study population of current smokers but not in currently smoking COPD cases only (Table 6). We adjusted for case-control status in models for FTND in the full set of currently smoking case and control subjects, and significant associations remained (p = 0.045 for rs8034191 and p = 0.01 for rs1051730).

**Table 6:** Multivariate analysis for the association with SNPs of CHRNA3/5 and nicotine dependence and emphysema severity in current smokers and ex-smokers

SNP (Chr. 15)				CHRNA3/5					
				**rs8034191**			**rs1051730**		

FTND score*				Beta	SE	p	Beta	SE	p

Current smokers	Combined (312)			0.45	0.21	0.03	0.53	0.21	0.01
	COPD (146)			0.32	0.30	0.30	0.29	0.31	0.35
	Control(166)			0.51	0.28	0.07	0.66	0.28	0.02

Log (%emphysema)^†^									

Current smokers			Combined (312)	0.02	0.10	0.83	0.03	0.10	0.77
			COPD (146)	-0.005	0.15	0.98	0.01	0.15	0.93
			Control (166)	0.06	0.10	0.58	0.03	0.11	0.76
Ex-smokers			Combined (681)	0.14	0.07	0.047	0.14	0.07	0.049
			COPD (344)	0.14	0.08	0.06	0.13	0.08	0.10
			Control (337)	-0.03	0.07	0.68	-0.02	0.07	0.75
Current and ex-smokers			Combined (985)	0.11	0.06	0.06	0.12	0.06	0.05
			COPD (490)	0.14	0.07	0.06	0.14	0.07	0.07
			Control (495)	-0.001	0.06	0.99	-0.002	0.06	0.98

* Adjusted for age and gender

^† ^Adjusted for age, gender, BMI, and CT scanner; current smokers included only the subjects with available FTND scores.

When the analysis was extended to include 681 COPD or control ex-smokers with genotyping data, both rs1051730 and rs8034191 showed significant associations with emphysema severity (p < 0.05); stratified analysis suggested that this association was likely being driven primarily by COPD subjects, since trends for association were observed in COPD subjects but not control subjects.

## Discussion

In this study, we described the relationships among nicotine dependence, a proven genetic susceptibility locus for nicotine dependence and COPD, and structural measures of COPD, including severity of emphysema and air-trapping on chest CT in COPD and non-COPD smoking controls. Although recently there was a report on the association of a SNP in the nAChR gene with emphysema severity [18], to our knowledge, this is the first analysis of the relationship among these three conditions in current smokers. Compatible with previous reports, FTND scores in our study population were correlated with the cumulative intensity of smoking in pack-years, daily amount of smoking, and younger age of smoking initiation [27,28]. In addition, the cumulative intensity of smoking in pack-years was correlated with lower lung function and emphysema. Nevertheless, contrary to our initial hypothesis, FTND score was negatively correlated with emphysema severity in both COPD and control subjects. In addition, FTND score decreased as COPD severity, assessed by GOLD stage, increased. We observed significant associations of rs8034191 and rs1051730 in the CHRNA3/5 locus with FTND score, but we found differential evidence for association of SNPs related to nicotine dependence with emphysema severity according to current smoking status.

Although nicotine was reported to inhibit apoptosis through the muscarinic acetylcholine receptor in some cell lines [29-31], any beneficial effect of current smoking on emphysema is extremely unlikely. There are several possible explanations for our finding of less quantitative radiographic emphysema and less severe COPD in subjects with greater nicotine addiction. The first potential explanation is selection bias in the study population, namely the 'healthy smoker effect' and/or 'survivor effect'. Cigarette smoking causes functional impairments such as troublesome sputum and cough, dyspnea, decrease in exercise capacity, and increased risk of mortality in subjects with COPD. These negative effects provide incentive to quit smoking, which is greater in more severely affected individuals. Since FTND score could be measured only in current smokers, affected subjects who have quit smoking are not included in our analysis. The development of COPD and the progression to more severe COPD could also lead to a reduction in daily smoking intensity among those individuals that continue to smoke; since the number of cigarettes currently smoked per day is part of the FTND, efforts to taper smoking could appear as a reduction in nicotine dependence. The observation that COPD subjects had greater average cigarettes smoked per day but similar current cigarettes smoked per day compared to control subjects (Table 1) suggests that some reduction in smoking intensity among COPD subjects has occurred with disease development. Since the daily cigarette usage is only one component of the FTND score, however, the impact of reducing cigarettes smoked per day on our results is uncertain.

Premature death may also lead to the exclusion of advanced COPD cases with severe emphysema or gas trapping from this study. This possibility is supported by the fact that the proportion of advanced stages of COPD was relatively low in this study population, and ex-smokers showed more severe obstruction on spirometry (Additional file 1; Table S2). The FTND may not reflect every aspect of nicotine dependence [32] and does likely have limitations in assessing nicotine dependence cross-sectionally in a smoking group of COPD subjects. Nevertheless, FTND has been validated for its usefulness in general population samples [20,21,32] and is widely used in studies including COPD populations [33,34]. Furthermore, in this study, the negative correlation between FTND and emphysema severity was found even in controls without airflow obstruction and in cases with mild-to-moderate COPD. Therefore, selection bias and the limitations of applying the FTND in COPD patients, likely do not explain all of our results.

Another plausible explanation for the negative association of FTND score with emphysema is interference in measuring the radiographic outcome variable of percent emphysema on quantitative chest CT scan analysis. Smoking induces airway irritation and inflammation and results in accumulation of mucus and inflammatory cells including neutrophils, macrophages, and lymphocytes in small airways even in subjects without airflow obstruction [35-37]. A previous study has shown that the count of inflammatory cells within small airway walls is correlated with the smoking intensity in pack-years and is higher in current than ex-smokers [35]. Camiciottoli *et al. *also reported that emphysema severity was higher in former smokers than in current smokers [38] as we observed in our study. Therefore, since subjects with higher dependence on nicotine tended to have greater current smoking exposures, those with higher FTND likely had more inflammatory changes in peripheral airways and alveoli which could be associated with increased lung density. Supporting this hypothesis, the effect of FTND on emphysema severity in multivariate analysis decreased and the effect on gas trapping disappeared after adjusting for the current number of cigarettes smoked per day. In addition, the association of nicotine addiction risk alleles with emphysema severity changed toward significant relationships in ex-smokers. Therefore, the hypothesis that measuring emphysema severity on chest CT might have limitations in current smokers may explain the differential genotypic association of SNPs with emphysema severity on chest CT according to their smoking status. The detection of significant genetic associations to quantitative CT emphysema phenotypes may be limited in current smokers due to this potential effect of current smoking on lung inflammation.

In smokers' lungs, emphysema is not the only possible phenotype and other pathological processes may increase lung density, including interstitial lung disease or other subclinical parenchymal diseases [39,40]. Lederer *et al *reported an increase of high attenuation areas with an increasing of amount of smoking even in healthy smokers [39]. This may also be the case in our study population. Considering our findings and previous reports related to the effects of smoking on CT imaging, more studies are needed to clarify the clinical significance of measuring low lung attenuation in populations that include current smokers.

In this study, we failed to show an association of nicotine dependence candidate SNPs with the severity of emphysema in current smokers while a significant association of candidate SNPs with nicotine dependence was found. Contrary to our findings in current smokers, Lambrechts *et al*. reported recently that rs1051730, one of the SNPs that we tested in this study, was associated with the presence and severity of emphysema while they did not show association of these SNPs with nicotine dependence measured by the number of pack-years smoked instead of FTND [18]. Among the subjects in Lambrechts' study, current smokers comprised only 45.6% and 50.7% of their two cohorts, which may decrease the overall effect of measuring emphysema in current smokers. They also used visual estimation of emphysema in one population[18]. These factors may lead to the differences from the results of our study, which is supported by the finding that the association of candidate SNPs with emphysema severity tended to be significant in our study when genetic association analysis was applied only in ex-smokers.

The association of SNPs in the CHRNA3/5 locus with smoking behavior has been was widely reported [41-43], and our results confirmed it again. We also analyzed genotypes in only white control subjects and COPD subjects with definite airflow obstruction (GOLD stage II-IV) to limit population heterogeneity.

Despite interesting findings, our study has limitations. As mentioned above, the population of advanced COPD cases was small, and the analysis across GOLD stages was limited. Our study sample was relatively small for genetic association analysis, and we did not include a replication population. We performed some genetic association analyses in a combined set of cases and controls. The appropriate adjustment for potential bias in such analyses is uncertain [44,45]. We performed adjustment for case-control status for FTND, but not for traits directly related to COPD pathophysiology (e.g. FEV1, emphysema) since this would likely have been an overadjustment. Although these are limitations, they are mitigated by the large body of evidence supporting a role for these SNPs in nicotine dependence in other populations.

In summary, FTND score was negatively associated with the severity of emphysema in COPD and healthy current smokers, and the FTND score decreased with increasing GOLD stage. Genetic variants in CHRNA3/5 (rs8034191 and rs1051730) were significantly associated with nicotine dependence. However, in a relatively small group of current smokers, an association of genetic variants in CHRNA3/5 (rs8034191 and rs1051730) with severity of emphysema or air trapping on CT was not found; the impact of current smoking on CT-measured emphysema may limit detection of significant genetic associations.

## Conclusion

Increased inflammation in more highly addicted current smokers could influence the CT lung density distribution; further investigation of the clinical significance of these findings will be necessary. While SNPs in CHRNA3/5 were associated with nicotine dependence measured with FTND, surprisingly, nicotine dependence was a negative predictor for emphysema on CT in COPD patients and even in control smokers. An association of genetic variants in CHRNA3/5 (rs8034191 and rs1051730) with severity of emphysema was found in former smokers, but not in current smokers. Our results suggest that current smoking could limit detection of genetic determinants of quantitative emphysema. The impact of current smoking status and reduction of smoking intensity with increased COPD severity need to be considered in epidemiological, radiological, and genetic studies of the relationships between nicotine addiction and COPD.

## Competing interests

Edwin K. Silverman has received grant support and consulting fees from GlaxoSmithKline for studies of COPD genetics; he has also received honoraria and consulting fees from AstraZeneca. The other authors declare that they have no competing interest.

## Authors' contributions

DKK contributed to study concept and design, analysis and interpretation of data, statistical support, and writing/editing of the manuscript. CPH contributed to funding, study concept and design, data collection, analysis and interpretation of data, and editing of the manuscript. GRW contributed to data collection and editing of the manuscript. JEH and JRM participated in analysis and interpretation of data, statistical support, and editing of the manuscript. DAL and JDN participated in collecting data and editing the manuscript. JDC contributed to funding, data collection, and editing of the manuscript. EKS contributed to funding, study concept and design, data collection, analysis and interpretation of data, and editing of the manuscript. All authors read and approved the final manuscript.

## Supplementary Material

Additional file 1 **Epidemiology, radiology, and genetics of nicotine dependence in COPD**. This additional file contains two supplementary tables to show the distribution of FTND severity in the study population (Table S1) and the comparative results of subpopulations according to their smoking status (Table S2). One additional figure (Figure S1) shows the distribution of subjects with high nicotine dependence across COPD GOLD stages. Click here for file
