# Supplementary material for: Epidemiology, radiology, and genetics of nicotine dependence in COPD
Source: Respir Res. 2011 Jan 13;12(1):9. doi: 10.1186/1465-9921-12-9 (PMC3033825; doi:10.1186/1465-9921-12-9)

Epidemiology, radiology, and genetics of nicotine dependence in COPD

Deog Kyeom Kim, Craig P. Hersh, George R. Washko, John E. Hokanson, David A. Lynch, John D. Newell, James R. Murphy, James D. Crapo, Edwin K. Silverman, and the COPDGene Investigators

Online Data Supplement

Table S1. The distribution of FTND severity in study population

|  | Control (n=507) | COPD (n=335) | p |
| --- | --- | --- | --- |
| FTND score (mean ±SD) | 4.6±2.5 | 4.7±2.4 | 0.43 |
| Nicotine dependence (FTND score) |  |  |  |
| No (0) | 33(6.5) | 13(3.9) | 0.71 |
| Very low (1-2) | 73(14.4) | 53(15.8) |  |
| Low (3-4) | 134(26.4) | 88(26.3) |  |
| Medium (5) | 71(14.0) | 48(14.3) |  |
| High (6-7) | 132(26.0) | 91(27.2) |  |
| Very high(8-10) | 64(12.6) | 42(12.5) |  |

Table S2. The comparison of subpopulation according to the smoking status^*^

|  | Control (988) | |  | COPD (1050) | |  |
| --- | --- | --- | --- | --- | --- | --- |
| Variables | Current (n=507) | Ex-smokers (n=481) | p | Current (n=335) | Ex-smokers  (n=715) | p |
| Age, year | 53.8±6.9 | 62.2±8.8 | <.0001 | 59.2±7.8 | 66.6±7.5 | <.0001 |
| Male, n (%) | 276(54.4) | 210(43.7) | 0.001 | 185(55.2) | 358(50.1) | 0.12 |
| White, n (%) | 245(48.3) | 428(89.0) | <.0001 | 244(72.8) | 612(85.6) | <.0001 |
| BMI, kg/m^2^ | 28.4±5.9 | 29.1±5.9 | 0.09 | 27.1±5.8 | 28.5±6.3 | 0.001 |
| Smoking amount, pack years | 37.2±19.2 | 37.5±22.0 | 0.85 | 51.7±28.8 | 54.3±25.6 | 0.16 |
| Smoking starting age, year | 17.0±5.3 | 17.3±3.8 | 0.32 | 16.4±4.8 | 16.9±4.0 | 0.12 |
| BODE index | 0.58±0.92 | 0.25±0.79 | <.0001 | 2.6±2.0 | 3.4±2.1 | <.0001 |
| FEV1/FVC, ratio | 0.79±0.05 | 0.78±0.05 | 0.09 | 0.53±0.12 | 0.46±0.13 | <.0001 |
| FEV1, % of predicted | 97.6±12.3 | 98.5±11.6 | 0.26 | 55.2±16.4 | 46.0±18.2 | <.0001 |
| % Emphysema at -950 HU, % | 1.84±2.21 | 3.72±3.49 | <.0001 | 8.81±9.75 | 18.53±13.51 | <.0001 |
| % Gas trapping, % | 10.5±9.9 | 13.1±8.8 | <.0001 | 33.0±19.3 | 46.7±19.2 | <.0001 |
| Pi_10_ | 3.75±0.12 | 3.73±0.12 | 0.049 | 3.81±0.12 | 3.79±0.13 | 0.02 |
| ^*^Data listed in number (%) for frequency or mean ±standard deviation for quantitative variables. Subjects with GOLD 1 or unclassified were excluded and current smokers included only smokers with available FTND score. | | | | | | |

Figure legends

Figure S1. The distribution of subjects with high nicotine dependence across COPD GOLD stages.


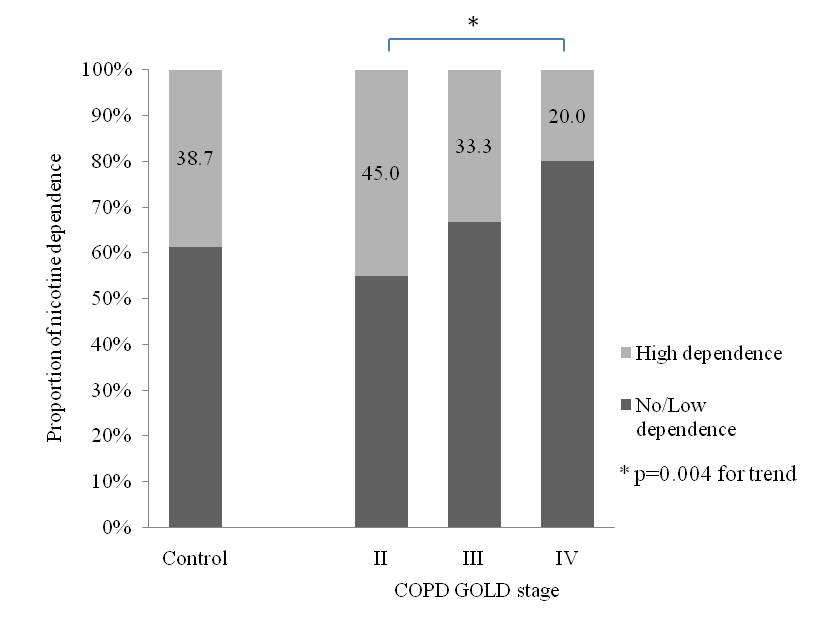

Supplement: Additional file 1 — Epidemiology, radiology, and genetics of nicotine dependence in COPD. This additional file contains two supplementary tables to show the distribution of FTND severity in the study population (Table S1) and the comparative results of subpopulations according to their smoking status (Table S2). One additional figure (Figure S1) shows the distribution of subjects with high nicotine dependence across COPD GOLD stages. [file 1465-9921-12-9-S1.DOC]
